# Enhancing Proprioceptive Input to Motoneurons Differentially Affects Expression of Neurotrophin 3 and Brain-Derived Neurotrophic Factor in Rat Hoffmann-Reflex Circuitry

**DOI:** 10.1371/journal.pone.0065937

**Published:** 2013-06-11

**Authors:** Olga Gajewska-Woźniak, Małgorzata Skup, Stefan Kasicki, Ewelina Ziemlińska, Julita Czarkowska-Bauch

**Affiliations:** Department of Neurophysiology, Nencki Institute of Experimental Biology, Warsaw, Poland; University of Sydney, Australia

## Abstract

The importance of neurotrophin 3 (NT-3) for motor control prompted us to ask the question whether direct electrical stimulation of low-threshold muscle afferents, strengthening the proprioceptive signaling, could effectively increase the endogenous pool of this neurotrophin and its receptor TrkC in the Hoffmann-reflex (H-reflex) circuitry. The effects were compared with those of brain-derived neurotrophic factor (BDNF) and its TrkB receptor. Continuous bursts of stimuli were delivered unilaterally for seven days, 80 min daily, by means of a cuff-electrode implanted over the tibial nerve in awake rats. The H-reflex was recorded in the soleus muscle to control the strength of stimulation. Stimulation aimed at activation of Ia fibers produced a strong increase of NT-3 protein, measured with ELISA, in the lumbar L3-6 segments of the spinal cord and in the soleus muscle. This stimulation exerted much weaker effect on BDNF protein level which slightly increased only in L3-6 segments of the spinal cord. Increased protein level of NT-3 and BDNF corresponded to the changes of NT-3 mRNA and BDNF mRNA expression in L3-6 segments but not in the soleus muscle. We disclosed tissue-specificity of TrkC mRNA and TrkB mRNA responses. In the spinal cord TrkC and TrkB transcripts tended to decrease, whereas in the soleus muscle TrkB mRNA decreased and TrkC mRNA expression strongly increased, suggesting that stimulation of Ia fibers leads to sensitization of the soleus muscle to NT-3 signaling. The possibility of increasing NT-3/TrkC signaling in the neuromuscular system, with minor effects on BDNF/TrkB signaling, by means of low-threshold electrical stimulation of peripheral nerves, which in humans might be applied in non-invasive way, offers an attractive therapeutic tool.

## Introduction

The role of neurotrophins in neuronal plasticity, particularly that involved in the recovery processes following injury of the spinal cord and peripheral nerves is well documented (for review see [1]). Neurotrophin 3 (NT-3) is indispensible for the development of muscle and tendon receptors. The relationship between NT-3 and the functional efficiency of proprioceptive systems has been demonstrated using NT-3 knockout mice which do not develop proper proprioceptive innervation and die shortly after birth [2]–[5].

In the adulthood, muscle receptors as well as the nerve fibers innervating them require NT-3 to recover after damage [2]–[4], [6]–[9]. In line with this requirement, about 73% of neurons innervating muscle spindle receptors show expression of TrkC mRNA [10]. A robust NT-3 mRNA expression was observed in intrafusal [6] and, to a lesser extent, in extrafusal muscle fibers [11] whereas TrkC mRNA expression was detected in skeletal muscles, predominantly in perisynaptic and myelinating Schwann cells [11]. In the dorsal root ganglia (DRG) the data on NT-3 mRNA expression are conflicting [12], [13] albeit recent study [13] reported on NT-3 mRNA and protein expression in DRG with a predominance in large neurons. Finally, NT-3 is expressed by numerous cells in the spinal cord, including motoneurons, interneurons, astro- and oligodendrocytes [14]. Trk C mRNA and protein were observed in neurons throughout the spinal grey matter including motoneurons of various size [15], [16].

Among various methods aimed at enriching damaged nervous tissue with neurotrophins (for review see [1], [17]), we have focused on those increasing endogenous pools of neurotrophins, assuming that their sources might be physiologically controlled and adapted to the local requirements [18]–[20].

The importance of NT-3 for motor control prompted us to ask whether direct electrical stimulation of low-threshold muscle afferents, targeted specifically to activation of nerve fibers conveying proprioceptive signals, could effectively increase the endogenous pool of this neurotrophin and its receptor in the spinal cord and muscles. The possibility of strengthening proprioceptive input to a selected group of motoneurons is of clear importance as recently there is an accumulation of experimental data indicating differentiated vulnerability of groups of neurons to the damage of the spinal cord [21], [22].

To address this question, we applied chronic electrical stimulation of low-threshold muscle afferent fibers of the tibial nerve, as their activation can produce physiologically relevant and controllable input to the motoneurons of ankle extensors in awake animals, recorded as a compound muscle action potential - Hoffmann (H) reflex, an analog of the monosynaptic stretch reflex. The amplitudes of H-reflexes and their accompanying direct motor responses (M) were monitored in the soleus muscle to control the strength of applied stimuli.

Low-threshold muscle afferent fibers (group Ia) terminate monosynaptically on their own motoneurons as well as on the motoneurons innervating muscles acting synergistically at the same and other joints [23]. Their direct input corresponds to several percent of the total synaptic input to motoneurons [24] but Ia afferents also affect motoneuron activity indirectly as they terminate on a number of spinal excitatory and inhibitory Ia and other types of interneurons, acting in concert with other peripheral, spinal and supraspinal inputs converging on these interneurons [25]–[27]. Therefore, chronic stimulation of the tibial nerve might not only reinforce Ia afferent input to the extensor motoneurons innervated by this nerve branch but also exert their effect through different groups of spinal interneurons. Moreover, as the majority of Ia boutons terminate on the dendrites of motoneurons, the afferent signal they convey is subjected to powerful amplification via the mechanisms of persistent inward current (PIC) present in these dendrites [28], [29].

Our assumption was that if expression of NT-3 and its high-affinity receptor, TrkC, are regulated in an activity-dependent mode (i.e., similarly as it was shown for the brain-derived neurotrophic factor (BDNF) and its high-affinity receptor TrkB) then chronic electrical stimulation of low-threshold muscle afferent fibers of the tibial nerve, dependent on NT-3, should primarily affect the expression of NT-3/TrkC in the circuitry of the monosynaptic H-reflex targeted to the motoneurons innervating the ankle extensor muscles and their synergists acting in other joints.

Here we show that seven days of electrical stimulation of low-threshold muscle afferent fibers of the tibial nerve by continuous bursts of pulses produced H-reflexes accompanied by near threshold direct motor responses, confirming that the stimulation was primarily targeted to group Ia afferent fibers. Chronic stimulation clearly increased the level of NT-3 protein, measured using ELISA, in the H-reflex circuitry. The effect was found both in lumbar (L3-6) segments of the spinal cord, where motoneurons innervating the majority of hindlimb muscles are located, and in the soleus muscle. Stimulation exerted a much weaker effect on the level of BDNF protein, which increased significantly only in the L3-6 segments of the spinal cord. Surprisingly, the effect of stimulation on NT-3 protein level in the circuitry of the H-reflex did not correspond to the changes of NT-3 mRNA expression but was accompanied by a profound increase of TrkC mRNA expression in the soleus muscle.

## Materials and Methods

### Animals

The experiments were carried out on 22 adult male Wistar rats weighting 280–350 g at the beginning of the experiments. These were divided into two groups: (1) intact control (N = 7), and (2) subjected to bilateral implantation of the muscle and nerve electrodes and to unilateral electrical stimulation of low-threshold muscle afferents in the tibial nerve (N = 15). The animals were bred in the animal house at the Nencki Institute, Warsaw, Poland. They were given free access to water and pellet food and were housed under standard humidity and temperature conditions on a 12 h light/dark cycle.

Experimental protocols involving animals, their surgery and care were approved by the First Local Ethics Committee in Warsaw and were in compliance with the guidelines of the European Community Council Directive 2010/63/UE of 22 September 2010 on the protection of animals used for scientific purposes.

### Implantation of Electrodes and Postsurgery Care

The animals were given subcutaneous injection of Butomidor (Butorfanolum, Richter Pharma, 1.5 mg/300 g b.w.) as a premedication and then anesthetized with isoflurane (Aerrane, Baxter, 1–2.5% in oxygen) via a facemask. Both hindlimbs and the back (low thoracic) were shaved and disinfected with 3% hydrogen peroxide at the incision sites at the popliteal fossa, over the soleus muscle and at the back. A connector plug for the electrodes was sewn to the muscles and ligaments over the vertebrae. The electrodes were drawn subcutaneously, bilaterally from the connector plug to the muscles and nerves to be implanted. For stimulation the pair of stainless-steel multistranded Teflon-coated fine- wires (Bergen Cable Technologies Inc., Lodi, NJ, U.S.A.) with final bare sewn into a silicone rubber cuff, as described by Loeb and Gans [30], was implanted over the tibial nerve, rostrally to the triceps surae branches. The tibial nerve was separated from the common peroneal and sural nerve branches in the popliteal fossa by means of fine microsurgical forceps with the use of magnifying glasses. The internal diameter of the cuff electrode was about 2-fold bigger than that of the tibial nerve. A compound muscle action potential was recorded in the soleus muscle by means of a pair of Teflon-coated fine-wire electrodes, with about 1.5 mm final bare, implanted into the soleus muscles so that the distance between the electrodes was about 5 mm. One set of electrodes was used for delivery of low-threshold stimulation to the tibial nerve to elicit a monosynaptic H-reflex recorded in the soleus muscle and the other, implanted on the contralateral side, served as a control of the effect of implantation (sham side). After the surgery, Baytril (Enrofloxacinum, 5 mg/kg, Bayer) was administered subcutaneously (s.c.) over five consecutive days to prevent infection. An analgesic Tolfedine (Tolfenamic acid 4%, 4 mg/kg, s.c. Vetoquinol S.A.) was given during the first three postoperative days. Immediately after the surgery, the rats were placed in warm cages, covered with blankets and inspected until fully awaken. Thereafter they were returned to individual cages with full access to food and water.

### Behavioral Training

In awake animals the amplitude of the H-reflex is highly modulated by the behavioral context and ongoing motor activity. Therefore, special care was taken to accustom the animals to being restrained in the immobilizing apparatus.

Two weeks before the stimulation experiment was initiated (one week after electrode implantation) the animals started to be accustomed to sitting in the restraining apparatus. The duration of every session was gradually prolonged until they reached 20 min criterion. Eighty min period of daily stimulation was divided into four 20 min sessions with about one hour rest in between. After every session the animals were rewarded with corn cookies in their home cages.

### Stimulation and H-reflex Recording

**Figure 1:**
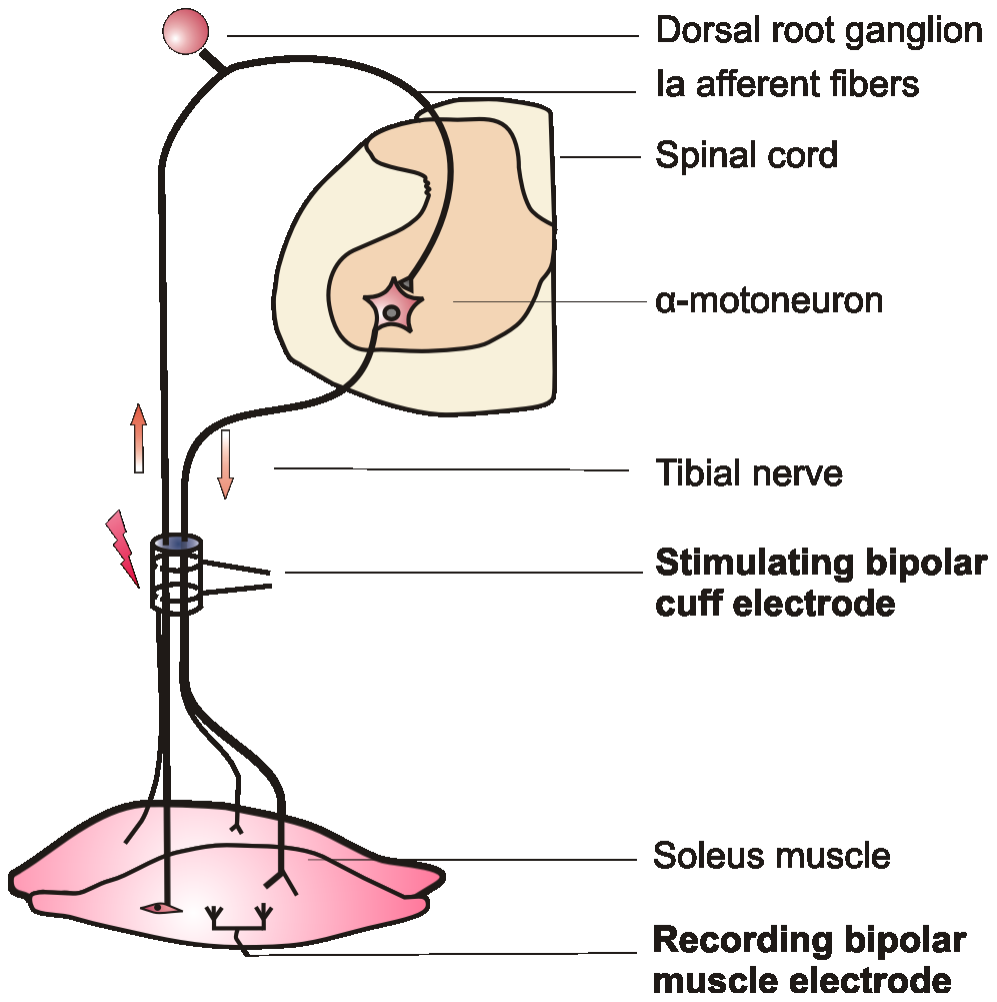
Schema showing the circuitry of the monosynaptic Hoffmann (H) reflex (an analog of the muscle stretch reflex) showing the location of the stimulating and recording electrodes. Low-threshold continuous burst stimulation of the tibial nerve, addressed to group Ia fibers, was applied to enhance the proprioceptive input to the extensor motoneurons innervating the ankle muscles. The amplitudes of H-reflex and direct M-response were used to control the strength of stimulation.

The monosynaptic H-reflex, an analog of the stretch reflex, was elicited by electrical stimulation of low-threshold muscle afferents (group Ia) in the tibial nerve and recorded as a compound muscle action potential in the soleus muscle (Figure 1). The strength of stimulation was established near the threshold of excitation of the motor fibers, which is higher than that activating Ia afferents, therefore it elicited a moderate H-reflex since the majority of Ia fibers are already excited when the direct motor response (M) is at its threshold [31], [Czarkowska-Bauch, unpublished].

The stimulus pattern consisted of continuous bursts of stimuli delivered every 25 ms for twenty minutes, 4 times per day. Each burst was composed of three pulses (pulse width = 200 µs) with 4 ms inter-pulse interval. This pattern of stimulation was chosen as it was expected to increase the probability of reflex responses of motoneurons under study and was applied in 10 animals [32]. The tibial nerve was stimulated unilaterally for seven days. Before and after daily stimulation sessions thirty H-reflexes elicited by single pulses (pulse width was 300 µs, at 0.3 Hz), were recorded to evaluate the effect of the continuous burst stimulation on the H-reflex amplitude. About 10 maximal direct motor responses (M_max_) elicited by single-pulse stimuli were collected in each animal to establish a reference value corresponding to the antidromic potential produced by all motoneurons in the soleus pool. The results concerning the area and/or amplitude of the H-reflexes and M-responses were expressed as a percentage of M_max_. Five animals were stimulated for 7 days by means of single stimuli (pulse width: 300 µs, at 0.3 Hz) to compare the effects of burst and single pulse stimulation on the probability of eliciting H-reflexes.

The stimulation experiments started about 3 weeks after implantation of the electrodes as only after that period the electrical conditions for eliciting the H-reflex have stabilized. Post-mortem inspection of the nerves and cuff electrodes showed that connective tissue filled the cuffs and stabilized the contact between the nerve and electrodes and increased the probability of eliciting H-reflexes.

### Data Acquisition and Analysis of Electrophysiological Data

**Figure 2:**
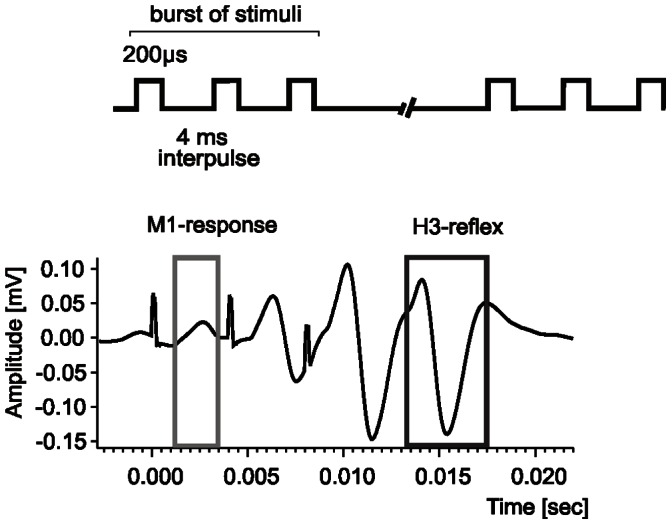
An example of averaged H- and M- waves in the soleus muscle elicited by continuous bursts of pulses applied to the tibial nerve every 25 ms. Each burst was composed of three pulses (pulse width = 200 µs) with 4 ms inter-pulse interval. The mean latencies (± SD) of the H-and M-responses recorded in the soleus muscle were 5.9±0.6 ms and 1.9±0.2 ms, respectively. During the burst stimulation only the first M_1_ and the last H_3_ response (framed) were analyzed as they were not contaminated by the accompanying responses.

The implanted connector plug was connected via a commutator to EMG amplifiers and to an isolated stimulation unit (Goeteborg Medical University workshop, Sweden). The analog signals were fed to a CED Micro 1401 ^mk II^ interface (Cambridge Electronic Design Ltd, UK), digitized and fed to a PC. Raw EMG activity was additionally monitored throughout the experiment on the oscilloscopes. A Spike 2 (Cambridge Electronic Design Ltd, UK) based script was used to measure the latency, peak-to-peak amplitude and the area of the H-reflex and M-response and to average these data. During continuous burst stimulation only the first M_1_ and the last H_3_ responses were taken into account as they were not contaminated by the accompanying responses (see Figure 2).

### Tissue Dissection for ELISA and Quantitative Real-time RT-PCR Analysis

One hour after the last stimulation session the rats were deeply anesthetized with a lethal dose of pentobarbital (80 mg/kg b.w., i.p.) and perfused transcardially with ice-cold saline. Next, the vertebral column was excised, placed on ice, spinal cord was removed in a cold room and frozen on dry ice. Cross-sections (0.8 mm thick) were cut on a McIlwain tissue chopper (Ted Pella Inc., Redding, CA, USA) and divided into left and right half (to compare the stimulated and sham side). Every second section was subjected to ELISA or to quantitative RT PCR (qRT PCR). Sections from L1-2 segments were analyzed separately from those collected from L3-6 segments. The soleus muscles were also sliced and subjected to ELISA or to qRT PCR for stimulated and sham side independently. The segments of the tibial nerve enwrapped in the cuff electrodes were subjected to qRT PCR analysis. All dissected tissues were stored at −80°C.

### Preparation of Homogenates for NT-3 and BDNF ELISA

For assessment of NT-3 protein level the *NT-3 Emax® ImmunoAssay System* (Promega Corporation, Madison, WI, USA) was used according to the manufacturer instructions. Crude tissue homogenates (10% w/v) were prepared in 20 mM Tris buffer (pH 8.0) containing 10% glycerol, 137 mM NaCl, 1% NP40, Complete*™* Protease Inhibitor Cocktail (Roche), PhosSTOP*™* Phosphatase Inhibitor Cocktail (Roche) with addition of 1 mM phenylmethyl-sulphonyl fluoride (PMSF; Sigma-Aldrich). An IKA Ultraturrax tissue grinder (for qRT PCR) or glass/glass grinder (for ELISA) were used to disrupt the tissue. The homogenates were incubated on ice for 30–60 min and centrifuged at 11 600×*g* for 30 min at 4°C. ELISA was performed on s1 supernates following the manufacturer’s instructions. All samples were run in triplicate. For BDNF ELISA (*ChemiKine™ BDNF Sandwich ELISA Kit*, Millipore, Billerica, MA, USA) a sample of tissue homogenates (10% w/v) prepared for NT-3 ELISA was complemented by adding a supplementary buffer which conformed the components of the lysis buffer to those recommended by the BDNF ELISA producer (100 mM Tris buffer pH 7.0, 2% BSA, 1 M NaCl, 2% Triton X-100 and Complete Protease Inhibitor Cocktail, 200 µM phenylmethyl-sulphonyl fluoride (PMSF; Sigma-Aldrich) and 157 µg/mL benzamidine hydrochloride (Serva, Heidelberg, Germany). Tissue incubation and centrifugation were carried out in the same conditions as in the NT-3 protocol. All samples were run in triplicate.

Protein content in the supernates was assayed by Bradford method [33].

### Analysis of NT3, TrkC, BDNF and TrkB Gene Expression by qRT PCR

Total RNA was isolated using ZR RNA MiniPrep™ kit (Zymo Research Corporation, Irvine, CA, USA) according to the manufacturer's protocol. After DNaseI treatment, total RNA (0.5–1 µg) was converted into coding DNA (cDNA). For the cDNA synthesis for spinal cord and muscle samples Transcriptor First Strand cDNA Synthesis Kit reagents (Roche Applied Science) and an anchored-oligo(dT)_18_ primers according to the manufacturer's protocol were used. Reverse transcription was performed at 50°C for 60 minutes followed by enzyme denaturation at 85°C for 5 minutes. For the tibial nerve samples, where the level of transcripts was very low, Transcriptor High Fidelity cDNA Synthesis Kit reagents (Roche Applied Science) and random hexamer primers were used according to the manufacturer's protocol. It increased efficiency in generation of complementary DNA for further qRT PCR analyses. The thermal cycling conditions for reverse transcription of these samples were programmed such that each sample cycled at 29°C for 10 minutes, at 48°C for 60 minutes, and at 85°C for 5 minutes. Quantification of NT3, BDNF, TrkC and TrkB gene transcripts was performed by means of TaqMan hydrolysis probes using the LightCycler ® 480 sequence detection system (Roche Applied Science, Indianapolis, IN, USA). Target-specific probes, forward and reverse primers as designed by Universal Probe Library Assay Design Center were used (Table S1 in the Supporting Information). Dual color qRT PCR was performed - each target gene transcript was analyzed in parallel with a probe specific for control transcript of glyceraldehyde-3-phosphate dehydrogenase (GAPDH) (Universal Probe Library Rat GAPD Gene Assay, Roche Applied Science). PCRs were run with cDNA equivalent to 50 ng of total RNA. Three-step PCR consisted of 10 s at 95°C, 30 sec. at 60°C and 1 s at 72°C (fluorescence acquisition step) for 45 to 55 cycles. LightCycler ® 480 Software 1.5.0 (Roche Applied Science) was used to determine the threshold cycle, in which the first significant increase of fluorescence occurred in individual amplifications. For each sample the threshold cycle values for target transcripts were automatically normalized to the simultaneously run GAPDH transcript amplification. The normalized values were used to make comparisons across experimental groups. Second derivative maximum algorithm was used for threshold cycle calculations. Also, by reason of duplex color PCR assay, a color compensation filter for data analyses was used.

For all target genes, TaqMan probes and primers were selected to amplify the regions which do not undergo alternative splicing. Thus they allow measuring a complete set of transcript variants (pan-probe/primer sets, Table S1 and Figure S1). A common feature of neurotrophin genes is that various types of transcripts are generated by alternative splicing of the 5′ short exons to the 3′ long exon encoding the entire protein. This is also true for the NT-3 gene. Because of the complexity of regulatory mechanisms of NT-3 expression and small number of reports on the expression patterns of the NT-3 transcript variants, in the present study we made an attempt to quantitatively assess four NT-3 gene transcript variants and estimate their contribution to the total NT-3 transcript pool (see Figure S1 and Table S1).

### Statistical Analysis

The non-parametric Mann-Whitney U test for comparison of independent samples, the *Sign* and Wilcoxon tests for comparison of related samples were used except for the analysis of the probability of eliciting H-reflexes elicited by the burst and single stimuli where a Student’s t-test was used. All tests were performed using *Statistica* software (StatSoft Inc, Tulsa, OK, USA).

## Results

### The Effect of Chronic Stimulation of Low-threshold Muscle Afferents on the Hoffmann Reflexes in the Soleus Muscle

The latencies of H- and M-responses were relatively stable in individual animals. The mean latency (± SD) of the H-reflex recorded in the soleus muscle was 5.9±0.6 ms whereas that of direct motor (M) response was 1.9±0.2 ms. The amplitude of the H-reflex recorded as a compound muscle action potential in awake animals was highly modulated by the behavioral context and ongoing motor activity during the experiment. Monitoring the direct motor (M) responses preceding the H-reflexes and setting the strength of the stimulation near the threshold for eliciting M-responses enabled delivery of the stimuli to low-threshold proprioceptive afferents (group Ia). This is because the majority of group Ia afferent fibers should be excited by the stimulation set near the threshold for eliciting M-responses [31].

**Figure 3:**
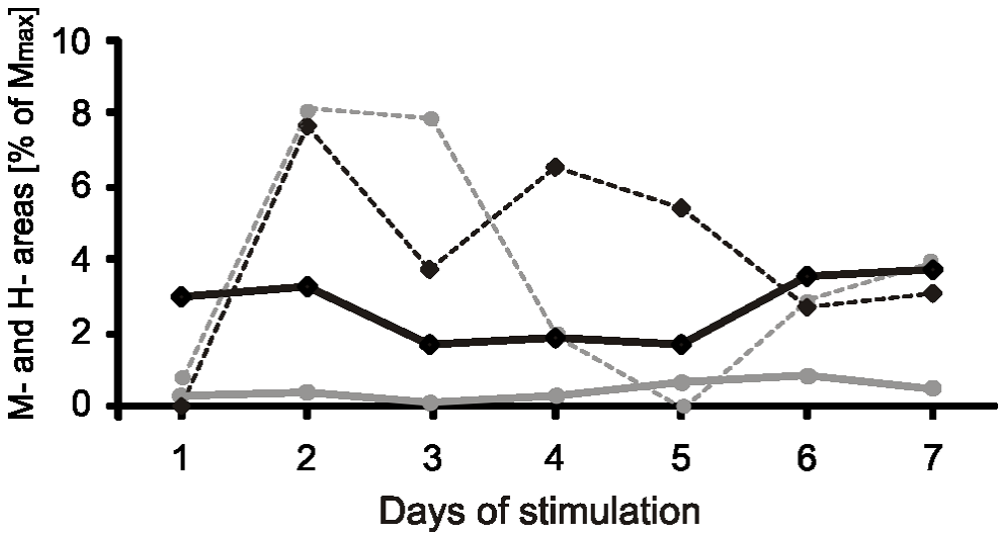
Time course of amplitude changes of the M- response and H- reflex evoked by electrical stimulation of the tibial nerve in the rat. Two stimulation patterns were used: continuous bursts of stimuli (solid lines: black – H reflex, grey – M response) or single stimuli (dashed lines: black - H reflex and grey – M response). The averaged responses to the single stimuli consisted of 30 reflexes collected at the beginning of the first and at the end of the last stimulation sessions daily in one animal. The averaged responses to burst of stimuli were collected during 3 min periods at the beginning and the end of every daily session. The strength of stimulation was established near the threshold of activation of the motor fibers, which is higher than that of Ia afferents, therefore stimulation elicited a moderate H-reflex as the majority of Ia fibers are already excited when the direct motor response (M) is at its threshold.

Figure 3 shows the changes of the area of H- and M-responses to the continuous bursts of stimuli compared to single stimuli during seven days of stimulation. It indicates that the H-reflex was usually accompanied by the near threshold M-response during continuous bursts stimulation creating much more stable conditions for eliciting H-reflexes than the single-pulse stimulation, where much higher variability of the responses was observed. When the areas of H-reflexes (averaged daily) elicited by single and burst stimuli were compared these differences were highly significant (*Sign* and Wilcoxon tests, P<0.0001) indicating that burst stimulation attenuated by about 50% the area of H-reflexes elicited by single pulses. On the other hand, the discharge probability of a motoneuron by means of high-frequency low-threshold continuous bursts of stimuli was higher than by single ones (Student t-test P<0.04).

We did not find any consistent effect of the order of daily sessions or the consecutive days of stimulation on the size of the H-reflexes (Sign and Wilcoxon tests, P>0.05).

Altogether, the H- and M-responses of the soleus to the continuous bursts of stimuli of the tibial nerve confirmed that it was possible to control, with this method, a delivery of the low-threshold proprioceptive input to the soleus motoneurons in order to evaluate its effect on the endogenous pool of NT-3 and its receptor TrkC in the spinal cord neurons, in the peripheral nerves and muscles involved in this monosynaptic reflex circuitry.

### NT-3 and BDNF Protein, their mRNA, TrkC mRNA and TrkB mRNA Levels in the H-reflex Circuitry in the intact Rat

**Figure 4:**
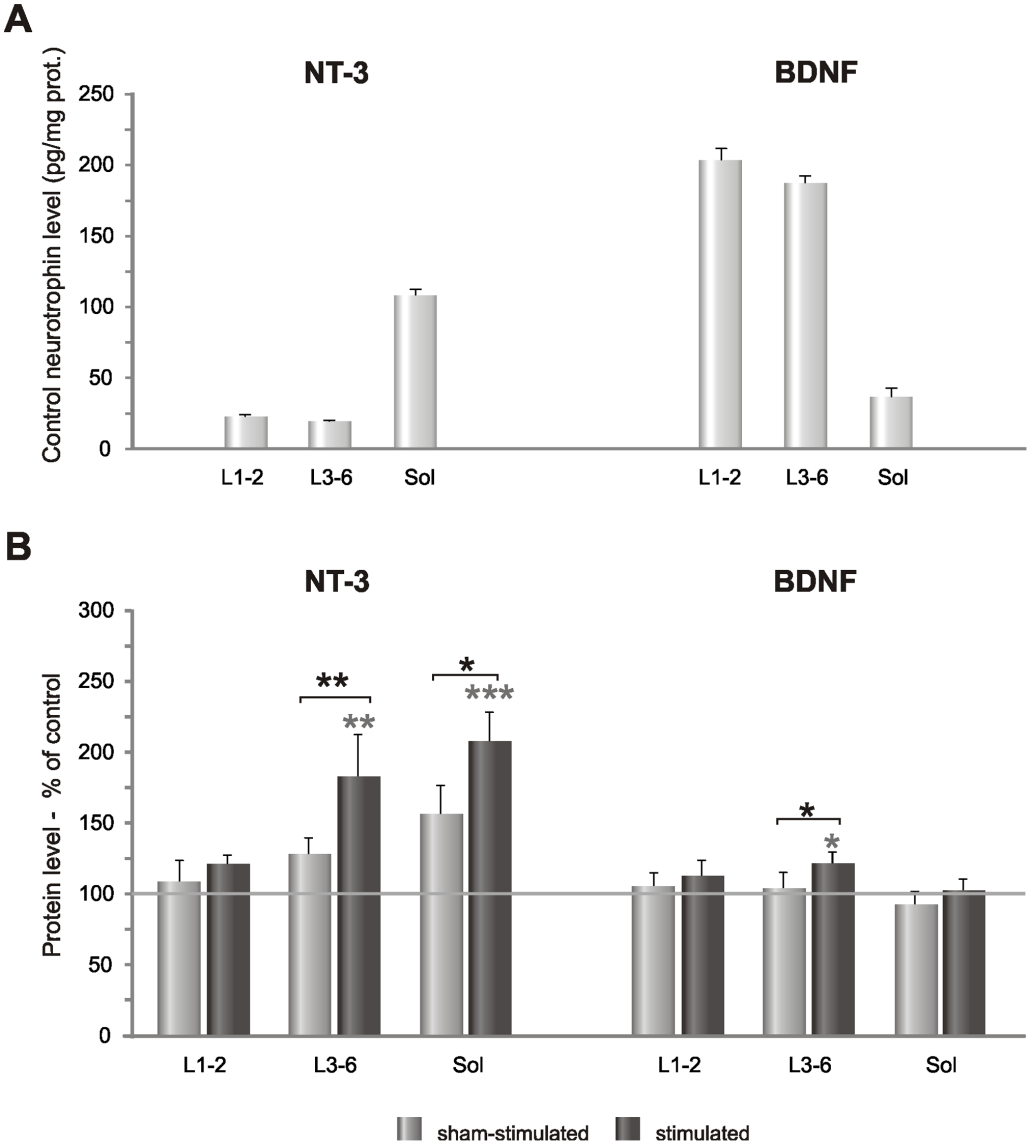
NT-3 and BDNF protein level in the tissues involved in the H-reflex circuitry. **A.** The protein level of NT-3 and BDNF in the intact animals (control group), measured in the lumbar segments (L1-2; L3-6) and the soleus muscle (sol), by means of ELISA. NT-3 level was the highest in the soleus muscle whereas that of BDNF predominated in the spinal cord. **B.** The changes of NT-3 and BDNF level in the spinal cord segments and soleus muscle after low-threshold, unilateral stimulation of the tibial nerve. Stimulation caused a clear increase of NT-3 protein level in the L3-6 segments of the spinal cord, where the motoneurons innervating the soleus muscles and its synergists acting at the ankle joints are located, and in the soleus muscle. The effect of stimulation on the protein level of BDNF was weaker and detected only in the caudal lumbar segments of the spinal cord. Asterisks indicate statistically significant effects (***P<0.001; **P<0.01; *P<0.05, Mann-Whitney U and Wilcoxon tests).

The protein level of NT-3 and BDNF in intact rats was different in each of the tissues involved in H-reflex circuitry (Figure 4 A). In the lumbar spinal cord NT-3 level was relatively low (over 20 pg/mg protein) but in the soleus muscle it was about 5 fold higher (over 100 pg/mg protein). Protein level of BDNF in the lumbar spinal cord (about 175 pg/mg protein) was, on the contrary, much higher than in the soleus muscle (about 50 pg/mg protein) (Figure 4 A).

**Figure 5:**
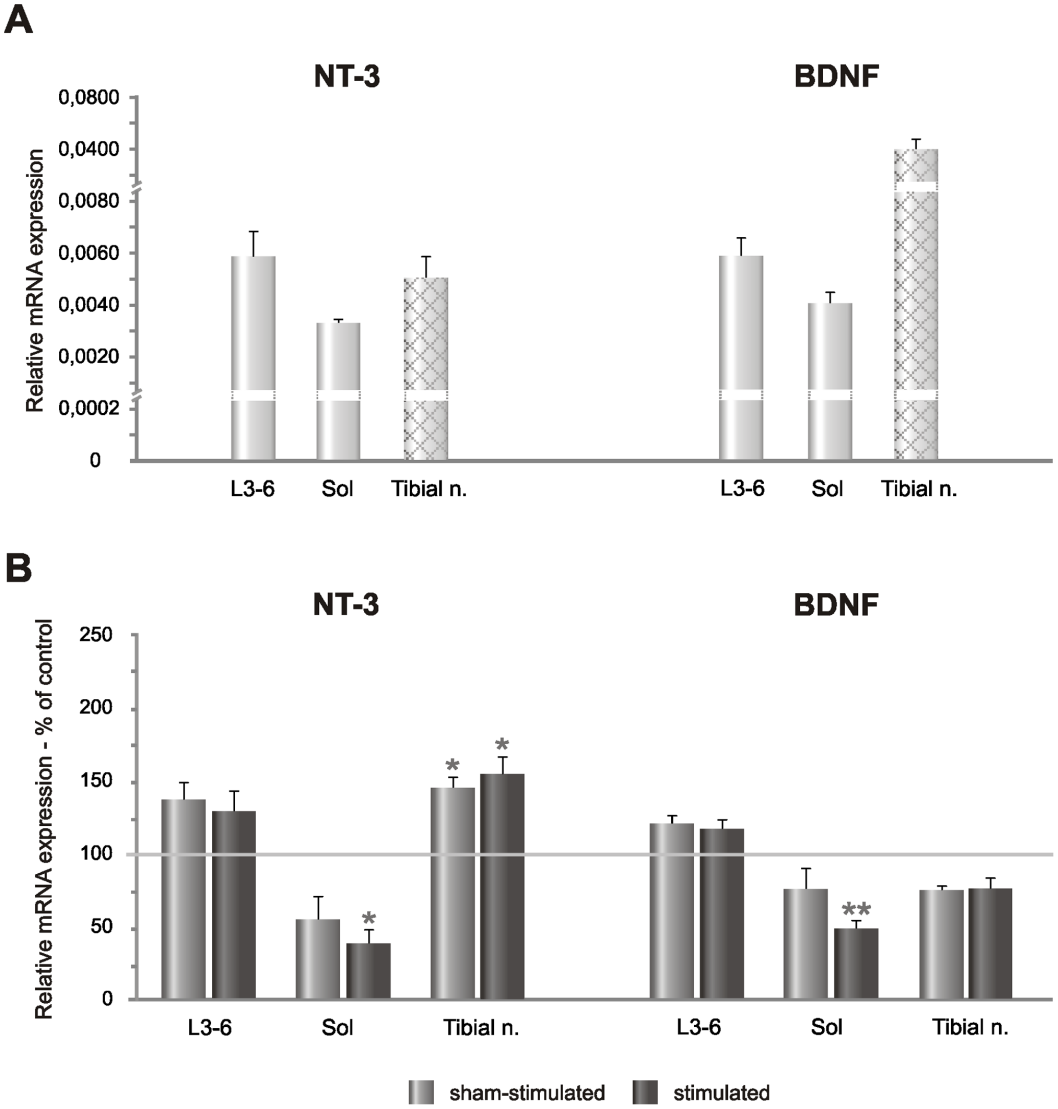
Distribution of NT-3 mRNA and BDNF mRNA expression in the tissues involved in circuitry of the H-reflex and their changes caused by continuous bursts of low-threshold, unilateral stimulation of the tibial nerve. **A.** Relative expression of transcripts in the control group. In the tibial nerve (shaded grey bars), mRNA expression was evaluated by means of high-fidelity reverse transcriptase and random hexamers as primers. The tibial nerve segment enwrapped in the cuff-electrode was analyzed. **B.** The changes of NT-3 and BDNF level in the spinal cord segments, soleus muscle and tibial nerve after low-threshold stimulation of the tibial nerve. NT-3 mRNA expression tended to increase in the caudal lumbar segments of the spinal cord and the effect was bilateral, similarly as that of BDNF mRNA. In the soleus muscle NT-3 mRNA and BDNF mRNA level decreased comparing to control level. NT-3 mRNA expression in the tibial nerve increased after stimulation but that of BDNF mRNA tended to decrease. Asterisks indicate statistically significant effects (***P<0.001; **P<0.01; *P<0.05, Mann-Whitney U and Wilcoxon tests).

In contrast to differences in the protein level of NT-3 and BDNF in the lumbar spinal cord and soleus muscle their mRNA expression was comparable in these tissues (Figure 5 A). Differences in the level of transcripts were observed in the tibial nerve where the expression of BDNF mRNA was 8 fold higher than of NT-3 mRNA (Figure 5 A).

**Figure 6:**
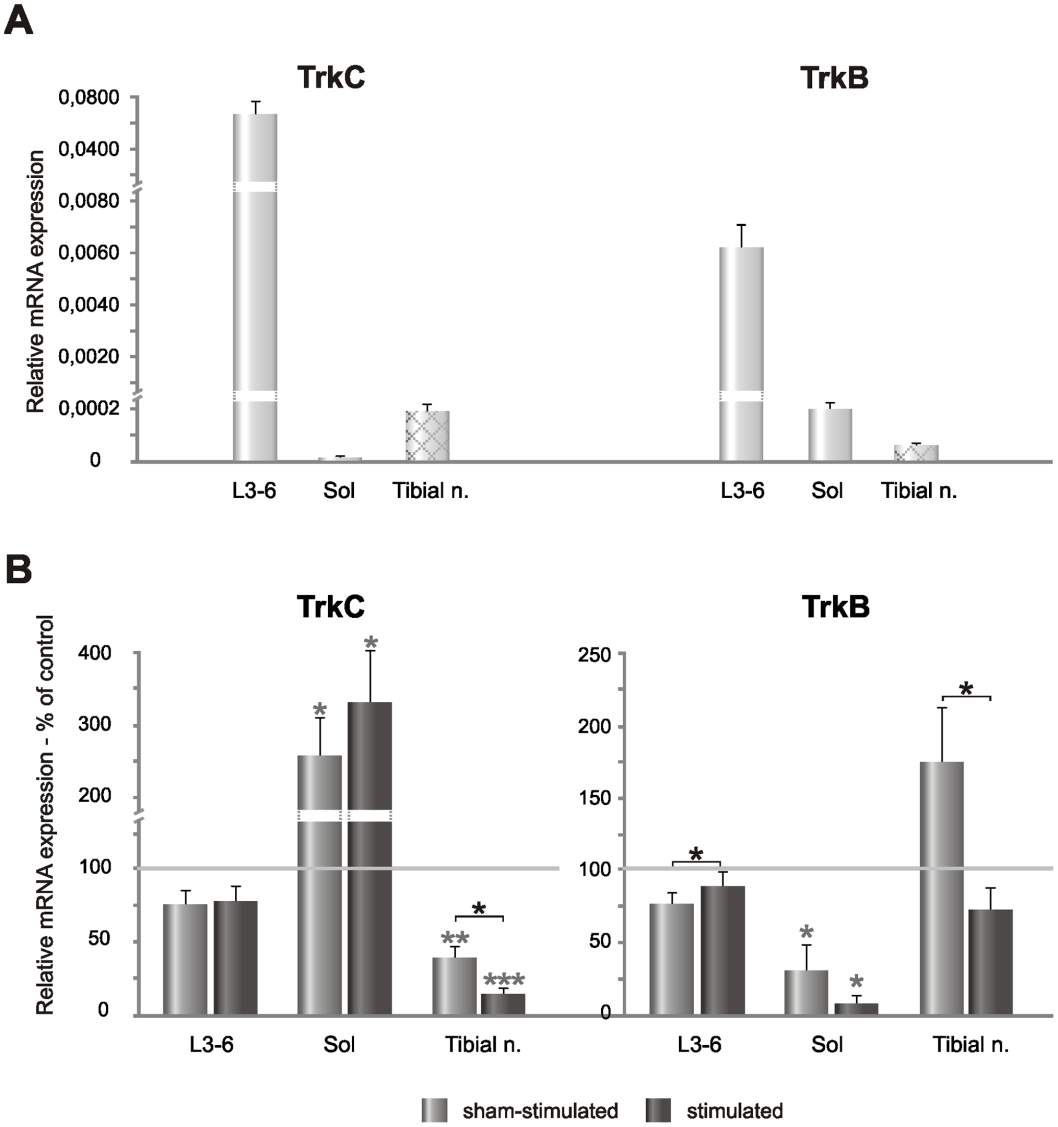
Distribution of TrkC and TrkB mRNA expression in the regions involved in H-reflex circuitry and their changes caused by continuous bursts of low-threshold, unilateral stimulation of the tibial nerve. **A.** Relative expression of transcripts in the control group. In the tibial nerve (shaded grey bars), the mRNA expression was evaluated by means of high-fidelity reverse transcriptase and random hexamers as primers. **B.** The changes of TrkC and TrkB level in the spinal cord segments, soleus muscle and tibial nerve after low-threshold stimulation. TrkC mRNA expression was high in the spinal cord of the control group and negligible in the soleus muscle. It tended to decrease after stimulation in the spinal cord and increased in the soleus muscle. Stimulation caused clear decrease of NT-3 mRNA in the tibial nerve. TrkB mRNA expression decreased both in the spinal cord and soleus muscle after stimulation but in the tibial nerve it increased on the sham-side and tended to decrease on the stimulated side. Asterisks indicate statistically significant effects (***P<0.001; **P<0.01; *P<0.05, Mann-Whitney U and Wilcoxon tests).

The expression of TrkC and TrkB mRNA was much more differentiated than of their ligands. TrkC mRNA expression was the highest in the L3-6 spinal segments, relatively low in the tibial nerve and negligible in the soleus muscle (Figure 6 A). The expression of TrkB mRNA was the highest in the spinal cord, low in the soleus muscle and the lowest in the tibial nerve (Figure 6 A). Although both TrkC and TrkB mRNA reached the highest level in the L3-6 spinal segments, the expression of TrkC mRNA was 10 fold higher than that of TrkB mRNA (Figure 6 A). Altogether, these results show clear differences between NT-3 and BDNF protein level in the tissues of the H-reflex circuitry which do not reflect their mRNA expression, suggesting that synthesis and transport of these two neurotrophins is differently regulated.

### Stimulation of Low-threshold Muscle Afferents causes an Increase of NT-3 Protein Level in the H-reflex Circuitry

Seven days of stimulation of low-threshold muscle afferents in the tibial nerve with the continuous burst pulses caused an increase of NT-3 protein level in L3-6 spinal segments by over 80% of the controls (Mann-Whitney U test, P = 0.006), and by over 40% comparing to the sham-stimulated hindlimb (Wilcoxon matched-pairs test, P = 0.01) causing no effect in L1-2 segments (Figure 4 B). A more pronounced increase was observed in the soleus muscle (by over 100% of the controls on the stimulated side (Mann-Whitney U test, P = 0.001) and by over 50% of that in sham-stimulated side (Wilcoxon matched-pairs, P = 0.03) (Figure 4 B).

### Changes of NT-3 mRNA and its High-affinity Receptor TrkC mRNA Expression after Stimulation

The pronounced increase of NT-3 protein level in L3-6 segments of the spinal cord with clear effect of stimulation was accompanied by a tendency to increase of NT-3 mRNA expression (measured by means of pan-probe no 73, detecting 1–4 transcript variants) by about 40% on the stimulated and by about 30% on the sham-side (Mann-Whitney U and Wilcoxon tests, non-significant – [n.s.]) (Figure 5 B). Surprisingly, in the soleus muscle, stimulation which increased NT-3 protein level caused a decrease of NT-3 mRNA expression by over 60% compared to intact rats (Mann-Whitney U test, P<0.01). Also on the sham-side NT-3 mRNA tended to decrease (by over 40%, n.s.) (Figure 5 B). This discrepancy led us to the more detailed analysis of the responses of different NT-3 transcripts with the use of other probes which showed that the decrease was owing to the response of the most abundant transcript 4, which was significantly down-regulated, whereas the smaller pool of transcripts 1–3 was increased (see Figure S1). In the tibial nerve NT-3 mRNA expression increased both on the stimulated and sham-side by 60% and 50%, respectively, compared to control values (Mann-Whitney U test, P = 0.04 and 0.03, respectively) (Figure 5).

The expression of TrkC mRNA in L3-6 segments of the spinal cord tended to decrease by over 20% (n.s.), both on the stimulated and sham-side, compared to control values (Figure 6 B). In contrast, there was over 3- and 2-fold increase of TrkC mRNA expression in the soleus muscle on the stimulated and sham-stimulated side, respectively (Mann-Whitney U test, P<0.01) (Figure 6 B). In the tibial nerve there was over an 80% decrease of TrkC transcript on the stimulated side (Mann-Whitney U, P<0.001) and over a 60% decrease on the sham-side comparing to control values (Mann-Whitney U, P<0.01). The effect of stimulation was also significant (Wilcoxon test, P<0.01) (Figure 6 B).

Overall, the effect of stimulation on the NT-3 mRNA and TrkC mRNA expression in the H-reflex circuitry was poorly differentiated between sides.

### Changes of BDNF Protein and Expression of BDNF mRNA and TrkB mRNA after Stimulation

The effect of stimulation on BDNF protein level was clearly weaker than on NT-3 and appeared only in L3-6 lumbar segments where it increased by 20% compared to control and sham values (Mann Whitney U and Wilcoxon tests, P<0.05) (Figure 4 B). BDNF mRNA expression in L3-6 spinal segments tended to increase by 20% (n.s.). In contrast to the BDNF protein level, its mRNA changed bilaterally (Figure 5 B). The opposite effect was found in the soleus muscle, where the stimulation caused approximately a 50% decrease of BDNF mRNA compared to control values (Mann Whitney U test, P = 0.005). On the sham-side there was also a tendency to decreased BDNF mRNA expression (n.s.) (Figure 5 B). BDNF mRNA expression tended to decrease also in the segment of the tibial nerve enwrapped in the cuff electrode where it was decreased by over 20% bilaterally, compared to control values (n.s.) (Figure 5 B).

TrkB mRNA expression decreased in L3-6 lumbar segments of the spinal cord by 13% on the stimulated- and by 25% on sham-side (Wilcoxon test, P<0.02). A dramatic decrease of TrkB mRNA expression was found in the soleus muscle (by about 93% on the stimulated and by about 70% on the sham-stimulated side) (Mann-Whitney U and Wilcoxon tests, P = 0.0003 and P = 0.014, respectively) (Figure 6 B). Stimulation tended to decrease TrkB mRNA expression in the tibial nerve by over 28% of the control values (n.s.) but it caused over a 2-fold increase in the sham-stimulated side (Figure 6 B) (Wilcoxon test, P = 0.05).

Overall, the stimulation-evoked a moderate increase of BDNF protein in the caudal lumbar segments was accompanied by the similar tendency in BDNF mRNA level. In the soleus muscle and the tibial nerve the same stimulation led to a decrease of BDNF mRNA and TrkB mRNA expression which did not translate into changes in BDNF protein.

## Discussion

The results of the present study demonstrate that chronic recording of the H-reflex from the soleus muscle elicited by low-threshold electrical stimuli targeted to Ia afferent fibers in the tibial nerve and monitoring the M-response enabled to control the strength of stimulation in awake rats. The strength of stimuli set at the threshold for the M-response was, if necessary, corrected down during stimulation, to limit the possibility of activation of higher threshold afferent fibers. We had rather risked missing the H-reflex than eliciting clear M-responses. However, a contamination of the H-reflex circuitry by activation of the group II/Aβ afferent fibers, even if minor, was possible in awake animals. Group II/Aβ afferents do not reach α-motoneurons directly but their activation could potentially affect the H-reflex elicited by the burst but not by single pulses. Therefore, one should expect changes of the H-reflex wave after burst stimulation due to bigger dispersion of incoming sensory volleys to the motoneuron pool. We did not find clear differences of the shape and duration of the H-reflexes elicited by single- or continuous burst pulses which would indicate a contamination of the H-reflex by additionally recruited afferent fibers of higher threshold than the group I afferents.

Continuous bursts of three stimuli were chosen to increase the probability of reflex responses of the motoneurons under study. Comparison of the H-reflexes elicited by single, low-frequency pulses and burst high-frequency stimuli confirmed that the latter increased the probability of eliciting the H-reflex, in agreement with the observations reported by Bawa and Chalmers [32] in humans. We also observed that bursts of low-threshold stimulation of the tibial nerve caused a depression of the amplitude of the H-reflexes compared to those elicited by single-pulses at 0.3 Hz. This effect, first described by Eccles and Rall, has been attributed to autogenic inhibition [34].

### Selectivity of NT-3 Protein Response to Low-threshold Stimulation of the Tibial Nerve

Seven days of continuous bursts of stimuli delivered in four 20 min sessions daily, separated by about 1 h breaks, clearly increased NT-3 protein level in L3-6 segments of the spinal cord and in the soleus muscle, without affecting the L1-2 segments of the spinal cord. On the sham-stimulated side there was a tendency for an increased level of NT-3 suggesting that the low-threshold stimuli used in this study activated monosynaptically not only α-motoneurons in the H-reflex circuitry but also interneurons distributing their axon terminals bilaterally (for reviews see [35], [36]). Similar bilateral effects were observed by Funakoshi and co-authors after 1 h of 50 Hz unilateral stimulation of the sciatic nerve in the acute conditions [37].

Low-threshold stimulation of the tibial nerve exerted a much weaker effect on the BDNF protein level, causing its increase in L3-6 segments of the spinal cord only. The differences between the responses of NT-3 and BDNF to low-threshold stimuli validated our concept. NT-3/TrkC is indispensable for the proprioceptive system, innervated predominantly by the thickest fibers in the peripheral nerves, of the highest conduction velocity and, by definition, having the lowest threshold of activation [23]. The BDNF/TrkB system is known to be involved in development and maintenance of the sensory system by controlling cutaneous mechanoreceptors [38], [39]. These fibers require a greater intensity of electrical stimuli to be activated than the group I proprioceptive fibers. Therefore, low-threshold stimuli used in our experiment were predominantly sub-threshold for the activation of the afferent fibers involved in cutaneous mechanosensation. Indeed, one hour of supramaximal 20 Hz stimulation of the cut and repaired femoral nerve caused over 2-fold increase of BDNF and TrkB mRNA in the motoneurons observed hours and days after stimulation [40].

### Opposite NT-3 mRNA and TrkC mRNA Response to Low-threshold Stimulation of the Tibial Nerve

The increased expression of BDNF compared to NT-3 transcripts in the tibial nerve, indicates that although both proteins are synthesized by Schwann cells, their functions and fate in the nerve are different. Namely, it suggests a dynamic state at which axonal translocation of BDNF towards target structures and NT-3 contribution to local actions on the Schwann cells prevail. Such interpretation is in line with data showing the importance of NT-3 as a major support factor for Schwann cells [41]. Loss of NT-3 *in vivo* produces degradation of Schwann cell functions, including myelination [42]. With this respect, a bilateral increase of NT-3 mRNA after stimulation may be interpreted as a generalized repair-directed response of Schwann cells to nerve implantation [43]. We may speculate that increased NT-3 leads to enhancement of local (autocrine and paracrine) functions but the net effect is difficult to predict as at the same time TrkC mRNA expression was down regulated.

Our data show that NT-3 mRNA and TrkC mRNA are differentially regulated. Opposite NT-3 mRNA and TrkC mRNA responses to electrical stimulation were also observed in hippocampal neurons [44], [45]. With all the reservations, and taking into account the strength and pattern of stimulation not comparable to our experimental paradigm, our data show that NT-3 mRNA and TrkC mRNA are differently regulated in various structures of the nervous system including neuromuscular system.

### A Mismatch of NT-3 mRNA and Protein Response to Stimulation in the Soleus Muscle

A discrepancy between the effect of stimulation on NT-3 protein and mRNA levels observed in soleus muscle in our study might stem from the different kinetics of mRNA and protein changes induced by stimulation. A one hour time lag between termination of the last stimulation session and decapitation was chosen as optimal for evaluation of protein and mRNA changes, based on our data on the stimulatory effect of long-term locomotor training on BDNF and NT-4 [18], [20], [46]. The studies cited above and the current data indicate a complex regulation of NT-3 expression. That complexity was disclosed in our study by showing that v1-v3 NT-3 transcripts respond to stimulation by an increase, matching an increase in the protein level, whereas the most abundant transcript 4 was decreased by stimulation (see Supporting Information). Verifying changes as a function of time would provide a more complete picture of alterations at the protein and mRNA level. Further studies elucidating the biological significance of generation of NT-3 transcripts, and in particular the abundant transcript 4 are needed.

### Possible Reasons for Protein and mRNA Incompatibility

Here, we provide evidence that NT-3 and BDNF- synthesizing cells and those producing TrkB and TrkC receptors respond to the stimulation differently in individual components of the H-reflex circuitry, as reflected by their differentiated mRNA expression. One of the differentiating factors stems from a type of rhythmical activation of H-reflex circuitry [18], [20], [47], [48]. For example, seven days of exercise in a running wheel increased NT-3 protein and mRNA expression as well as TrkC expression in the lumbar spinal cord [47], whereas 7 days of continuous bursts of low-threshold stimulation of the tibial nerve, increased the NT-3 protein response, but decreased TrkC mRNA. A more explicit difference was found in the soleus muscle where TrkC mRNA expression did not change after wheel-running exercise [47] but it dramatically increased after low-threshold electrical stimulation of the tibial nerve (this study).

The other reason of incompatibility may be the time lag between termination of the experiment and killing the animals. When NT-3 and BDNF mRNA expression was examined in the lumbar spinal cord and soleus muscle of intact rats subjected to 5 days of 30 min daily walking exercise on a moving treadmill belt, there was no immediate change in the lumbar spinal cord after the exercise, but 2 h later there was over a 50% increase of both neurotrophins [49]. On the other hand, in the soleus muscle both NT-3 and BDNF mRNA expression increased immediately after exercise [49]. Therefore, these data indicate that the time lag optimal for the evaluation of the mRNA expression was different in the spinal cord and muscle tissues, reflecting different dynamics of changes of these neurotrophins in H-reflex circuitry.

### The Effects of Chronic Implantation of Electrodes on the H-reflex Circuitry

We considered the possibility, that chronic cuff electrodes over the posterior tibial nerve and intramuscular electrodes in the soleus muscle used in our experiments could affect the results observed in the H-reflex circuitry. There are indications that some compression of the nerve enwrapped for several months in the cuff-electrodes may cause damage affecting the most vulnerable large diameter Ia fibers [50], [51]. However, our experiment lasted much shorter (4–5 weeks) and the probability that compression in the cuff developed is low as this process requires time after implantation [50]. Moreover, changes in NT-3 and BDNF protein levels and mRNA expression observed 28 days after mild compression of the sciatic nerve in the rats [52] do not match the changes observed in our study suggesting that chronic implantation of the cuff and intramuscular electrodes did not significantly affect the presented results.

### Physiological Implications of Strengthening of Low-threshold Proprioceptive Input to Motoneurons

Our study showed that low-threshold continuous bursts of electrical stimuli addressed to activation of the group Ia afferents offer an attractive possibility of enhancement of NT-3 signaling in the circuitry of the H-reflex. It poses the question on the physiological implications of low-threshold proprioceptive stimulation for motoneuron excitability. Muscle spindle-derived NT-3 was found to be necessary for strengthening connections between Ia afferents and motoneurons during the early postnatal period [53], [54]. However, moderately increased levels of NT-3 in the spinal cord caused a decrease of input resistance of motoneurons, an increase of their threshold for discharge and decline of amplitude of EPSPs to maximal monosynaptic Ia stimuli [17] indicating that in mature rats NT-3 might attenuate motoneuron excitability. BDNF exerts a different effect on the motoneuron excitability. When applied to the gastrocnemius muscle it increased input resistance of motoneurons [55]. Changes of excitability of the spinal neuronal network induced by BDNF or NT-3 in rats after spinal cord transection and intraspinal delivery of adeno-associated viral vectors carrying NT-3 or BDNF constructs confirmed the above observations [56], [57]. The increased level of NT-3 in the spinal cord after low-threshold stimulation observed in our experiments leaves open the question whether it also caused similar changes of excitability of the motoneurons. Interestingly, in the oculomotor system trophic support of axotomized fibers of abducens motoneurons caused restoration of phasic firing with NT-3 treatment and tonic firing with BDNF treatment [58]. The latter observations show another aspect of the motoneuron activity which might be modified by increased level of NT-3.

To conclude, as NT-3 exerts beneficial effects on damaged sensory fibers and motoneurons [59], plays an analgesic role [60] and is indispensable for the survival and differentiation of Schwann cells [46], an increase of NT-3 availability after injury or demyelinization of peripheral nerves may be of particular importance. The recent data show that attenuated expression of NT-3 may lead to proapoptotic signaling through TrkC receptor, which in experimental conditions behaves as a dependence receptor and, as such, induces caspase-dependent apoptotic death in the absence of ligand [61]. Therefore, providing the possibility of increasing the NT-3 expression in the neuromuscular circuitry by means of low-threshold electrical stimulation of peripheral nerves, which in humans might be applied in non-invasive way, offers an attractive therapeutic tool.

## Supporting Information

Figure S1 **Contribution of NT-3 transcript variants to the total pool of NT-3 mRNA in the soleus muscle.** **A.** Maps of 4 variants of NT-3 transcripts. Triangles point to sequences recognized by individual probes used in qPCR analysis. **B.** In the intact controls the level of the transcript variant 4 was 30 times higher than the level of three remaining variants (compare the results for probe #73 and #29). **C.** Electrical stimulation of the tibial nerve caused a significant down-regulation of the total mRNA level (probe #73) and similar tendency in the sham-stimulated side. A decrease is attributed to the abundant transcript variant 4 since three remaining variants (v1÷3) as well as variant 3 alone, analyzed separately, were significantly upregulated. **D.** By increasing the levels of v1÷3 transcripts and decreasing the level of v 4 transcript, electrical stimulation and sham-stimulation alters their contribution to an overall NT-3 mRNA pool.
(TIF)

Table S1 **Sequences of TaqMan probes and primers used for quantitative analysis of NT3, BDNF, TrkC and TrkB gene expression by qPCR.**
(DOC)

## References

[1] Lu P, Tuszynski MH (2008) Growth factors and combinatorial therapies for CNS regeneration. Exp Neurol 209: 313–320. 17927983 10.1016/j.expneurol.2007.08.004 PMC2408882

[2] Ernfors P, Lee KF, Kucera J, Jaenisch R (1994) Lack of neurotrophin-3 leads to deficiencies in the peripheral nervous system and loss of limb proprioceptive afferents. Cell 77: 503–512. 7514502 10.1016/0092-8674(94)90213-5

[3] Farinas I, Jones KR, Backus C, Wang XY, Reichardt LF (1994) Severe sensory and sympathetic deficits in mice lacking neurotrophin-3. Nature 369: 658–661. 8208292 10.1038/369658a0

[4] Klein R, Silos-Santiago I, Smeyne RJ, Lira SA, Brambilla R, et al (1994) Disruption of the neurotrophin-3 receptor gene trkC eliminates la muscle afferents and results in abnormal movements. Nature 368: 249–251. 8145824 10.1038/368249a0

[5] Wright DE, Zhou L, Kucera J, Snider WD (1997) Introduction of a neurotrophin-3 transgene into muscle selectively rescues proprioceptive neurons in mice lacking endogenous neurotrophin-3. Neuron 19: 503–517. 9331344 10.1016/s0896-6273(00)80367-0

[6] Copray JC, Brouwer N (1994) Selective expression of neurotrophin-3 messenger RNA in muscle spindles of the rat. Neuroscience 63: 1125–1135. 7700514 10.1016/0306-4522(94)90578-9

[7] Barbacid M (1994) The Trk family of neurotrophin receptors. J Neurobiol 25: 1386–1403. 7852993 10.1002/neu.480251107

[8] Bradbury EJ, Khemani S, Von R, King, Priestley JV, et al (1999) NT-3 promotes growth of lesioned adult rat sensory axons ascending in the dorsal columns of the spinal cord. Eur J Neurosci 11: 3873–3883. 10583476 10.1046/j.1460-9568.1999.00809.x

[9] Mendell LM, Johnson RD, Munson JB (1999) Neurotrophin modulation of the monosynaptic reflex after peripheral nerve transection. J Neurosci 19: 3162–3170. 10191329 10.1523/JNEUROSCI.19-08-03162.1999 PMC6782282

[10] McMahon SB, Armanini MP, Ling LH, Phillips HS (1994) Expression and coexpression of Trk receptors in subpopulations of adult primary sensory neurons projecting to identified peripheral targets. Neuron 12: 1161–1171. 7514427 10.1016/0896-6273(94)90323-9

[11] Hess DM, Scott MO, Potluri S, Pitts EV, Cisterni C, et al (2007) Localization of TrkC to Schwann cells and effects of neurotrophin-3 signaling at neuromuscular synapses. J Comp Neurol 501: 465–482. 17278135 10.1002/cne.21163

[12] Shen H, Chung JM, Chung K (1999) Expression of neurotrophin mRNAs in the dorsal root ganglion after spinal nerve injury. Brain Res Mol Brain Res 64: 186–192. 9931485 10.1016/s0169-328x(98)00314-3

[13] Wang TH, Meng QS, Qi JG, Zhang WM, Chen J, et al (2008) NT-3 expression in spared DRG and the associated spinal laminae as well as its anterograde transport in sensory neurons following removal of adjacent DRG in cats. Neurochem Res 33: 1–7. 17710544 10.1007/s11064-007-9398-6

[14] Riley CP, Cope TC, Buck CR (2004) CNS neurotrophins are biologically active and expressed by multiple cell types. J Mol Histol 35: 771–783. 15609090 10.1007/s10735-004-0778-9

[15] Merlio JP, Ernfors P, Jaber M, Persson H (1992) Molecular cloning of rat trkC and distribution of cells expressing messenger RNAs for members of the trk family in the rat central nervous system. Neuroscience 51: 513–532. 1488112 10.1016/0306-4522(92)90292-a

[16] Copray S, Kernell D (2000) Neurotrophins and trk-receptors in adult rat spinal motoneurons: differences related to cell size but not to 'slow/fast' specialization. Neurosci Lett 289: 217–220. 10961668 10.1016/s0304-3940(00)01305-7

[17] Petruska JC, Kitay B, Boyce VS, Kaspar BK, Pearse DD, et al (2010) Intramuscular AAV delivery of NT-3 alters synaptic transmission to motoneurons in adult rats. Eur J Neurosci 32: 997–1005. 20849530 10.1111/j.1460-9568.2010.07392.x PMC2943849

[18] Macias M, Dwornik A, Ziemlinska E, Fehr S, Schachner M, et al (2007) Locomotor exercise alters expression of pro-brain-derived neurotrophic factor, brain-derived neurotrophic factor and its receptor TrkB in the spinal cord of adult rats. Eur J Neurosci 25: 2425–2444. 17445239 10.1111/j.1460-9568.2007.05498.x

[19] Macias M, Nowicka D, Czupryn A, Sulejczak D, Skup M, et al (2009) Exercise-induced motor improvement after complete spinal cord transection and its relation to expression of brain-derived neurotrophic factor and presynaptic markers. BMC Neurosci 10: 144. 19961582 10.1186/1471-2202-10-144 PMC2802589

[20] Skup M, Dwornik A, Macias M, Sulejczak D, Wiater M, et al (2002) Long-term locomotor training up-regulates TrkB(FL) receptor-like proteins, brain-derived neurotrophic factor, and neurotrophin 4 with different topographies of expression in oligodendroglia and neurons in the spinal cord. Exp Neurol 176: 289–307. 12359171 10.1006/exnr.2002.7943

[21] Zagoraiou L, Akay T, Martin JF, Brownstone RM, Jessell TM, et al (2009) A cluster of cholinergic premotor interneurons modulates mouse locomotor activity. Neuron 64: 645–662. 20005822 10.1016/j.neuron.2009.10.017 PMC2891428

[22] Skup M, Gajewska-Wozniak O, Grygielewicz P, Mankovskaya T, Czarkowska-Bauch J (2012) Different effects of spinalization and locomotor training of spinal animals on cholinergic innervation of the soleus and tibialis anterior motoneurons. Eur J Neurosci 36: 2679–2688. 22708650 10.1111/j.1460-9568.2012.08182.x

[23] Laporte Y, Lloyd DP (1952) Nature and significance of the reflex connections established by large afferent fibers of muscular origin. Am J Physiol 169: 609–621. 14943853 10.1152/ajplegacy.1952.169.3.609

[24] Burke RE, Glenn LL (1996) Horseradish peroxidase study of the spatial and electrotonic distribution of group Ia synapses on type-identified ankle extensor motoneurons in the cat. J Comp Neurol 372: 465–485. 8873872 10.1002/(SICI)1096-9861(19960826)372:3<465::AID-CNE9>3.0.CO;2-0

[25] Jankowska E, Puczynska A (2008) Interneuronal activity in reflex pathways from group II muscle afferents is monitored by dorsal spinocerebellar tract neurons in the cat. J Neurosci 28: 3615–3622. 18385320 10.1523/JNEUROSCI.0466-08.2008 PMC6671081

[26] Czarkowska J, Jankowska E, Sybirska E (1976) Axonal projections of spinal interneurones excited by group I afferents in the cat, revealed by intracellular staining with horseradish peroxidase. Brain Res 118: 115–118. 990947 10.1016/0006-8993(76)90844-1

[27] Czarkowska J, Jankowska E, Sybirska E (1981) Common interneurones in reflex pathways from group 1a and 1b afferents of knee flexors and extensors in the cat. J Physiol 310: 367–380. 7230040 10.1113/jphysiol.1981.sp013555 PMC1274746

[28] Hyngstrom AS, Johnson MD, Miller JF, Heckman CJ (2007) Intrinsic electrical properties of spinal motoneurons vary with joint angle. Nat Neurosci 10: 363–369. 17293858 10.1038/nn1852

[29] Heckman CJ, Johnson M, Mottram C, Schuster J (2008) Persistent inward currents in spinal motoneurons and their influence on human motoneuron firing patterns. Neuroscientist 14: 264–275. 18381974 10.1177/1073858408314986 PMC3326417

[30] Loeb GE, Gans C (1986) Electromyography for experimentalists. University of Chicago Press.

[31] Schieppati M (1987) The Hoffmann reflex: a means of assessing spinal reflex excitability and its descending control in man. Prog Neurobiol 28: 345–376. 3588965 10.1016/0301-0082(87)90007-4

[32] Bawa P, Chalmers G (2008) Responses of Human Motoneurons to High-Frequency Stimulation of Ia Afferents. Muscle & Nerve 38: 1604–1615. 19016548 10.1002/mus.21184

[33] Bradford MM (1976) A rapid and sensitive method for the quantitation of microgram quantities of protein utilizing the principle of protein-dye binding. Anal Biochem 72: 248–254. 942051 10.1016/0003-2697(76)90527-3

[34] Eccles JC, Rall W (1951) Repetitive monosynaptic activation of motoneurones. Proc R Soc Lond B Biol Sci 138: 475–498. 14911805 10.1098/rspb.1951.0036

[35] Jankowska E (2008) Spinal interneuronal networks in the cat: elementary components. Brain Res Rev 57: 46–55. 17884173 10.1016/j.brainresrev.2007.06.022 PMC2683333

[36] Jankowska E (1992) Interneuronal relay in spinal pathways from proprioceptors. Prog Neurobiol 38: 335–378. 1315446 10.1016/0301-0082(92)90024-9

[37] Funakoshi H, Belluardo N, Arenas E, Yamamoto Y, Casabona A, et al (1995) Muscle-derived neurotrophin-4 as an activity-dependent trophic signal for adult motor neurons. Science 268: 1495–1499. 7770776 10.1126/science.7770776

[38] Carroll P, Lewin GR, Koltzenburg M, Toyka KV, Thoenen H (1998) A role for BDNF in mechanosensation. Nat Neurosci 1: 42–46. 10195107 10.1038/242

[39] Gonzalez-Martinez T, Farinas I, Del Valle ME, Feito J, Germana G, et al (2005) BDNF, but not NT-4, is necessary for normal development of Meissner corpuscles. Neurosci Lett 377: 12–15. 15722178 10.1016/j.neulet.2004.11.078

[40] Al-Majed AA, Brushart TM, Gordon T (2000) Electrical stimulation accelerates and increases expression of BDNF and trkB mRNA in regenerating rat femoral motoneurons. Eur J Neurosci 12: 4381–4390. 11122348

[41] Meier C, Parmantier E, Brennan A, Mirsky R, Jessen KR (1999) Developing Schwann cells acquire the ability to survive without axons by establishing an autocrine circuit involving insulin-like growth factor, neurotrophin-3, and platelet-derived growth factor-BB. J Neurosci 19: 3847–3859. 10234017 10.1523/JNEUROSCI.19-10-03847.1999 PMC6782711

[42] Woolley AG, Tait KJ, Hurren BJ, Fisher L, Sheard PW, et al (2008) Developmental loss of NT-3 in vivo results in reduced levels of myelin-specific proteins, a reduced extent of myelination and increased apoptosis of Schwann cells. Glia 56: 306–317. 18080292 10.1002/glia.20614

[43] Gajewska-Wozniak O, Skup M, Kasicki S, Czarkowska-Bauch J (2012) Low-threshold stimulation of tibial nerve causes profound increase of neurotrophin 3 but not BDNF in the spinal cord and muscles. FENS Abstracts. Barcelona.

[44] Kokaia Z, Kelly ME, Elmer E, Kokaia M, McIntyre DC, et al (1996) Seizure-induced differential expression of messenger RNAs for neurotrophins and their receptors in genetically fast and slow kindling rats. Neuroscience 75: 197–207. 8923534 10.1016/0306-4522(96)00257-6

[45] Elmer E, Kokaia M, Kokaia Z, Ferencz I, Lindvall O (1996) Delayed kindling development after rapidly recurring seizures: relation to mossy fiber sprouting and neurotrophin, GAP-43 and dynorphin gene expression. Brain Res 712: 19–34. 8705303 10.1016/0006-8993(95)01424-1

[46] Macias M, Fehr S, Dwornik A, Sulejczak D, Wiater M, et al (2002) Exercise increases mRNA levels for adhesion molecules N-CAM and L1 correlating with BDNF response. Neuroreport 13: 2527–2530. 12499861 10.1097/00001756-200212200-00029

[47] Ying Z, Roy RR, Edgerton VR, Gomez-Pinilla F (2003) Voluntary exercise increases neurotrophin-3 and its receptor TrkC in the spinal cord. Brain Res 987: 93–99. 14499950 10.1016/s0006-8993(03)03258-x

[48] Cote MP, Azzam GA, Lemay MA, Zhukareva V, Houle JD (2011) Activity-dependent increase in neurotrophic factors is associated with an enhanced modulation of spinal reflexes after spinal cord injury. J Neurotrauma 28: 299–309. 21083432 10.1089/neu.2010.1594 PMC3037803

[49] Gomez-Pinilla F, Ying Z, Opazo P, Roy RR, Edgerton VR (2001) Differential regulation by exercise of BDNF and NT-3 in rat spinal cord and skeletal muscle. Eur J Neurosci 13: 1078–1084. 11285004 10.1046/j.0953-816x.2001.01484.x

[50] Carp JS, Chen XY, Sheikh H, Wolpaw JR (2001) Operant conditioning of rat H-reflex affects motoneuron axonal conduction velocity. Exp Brain Res 136: 269–273. 11206290 10.1007/s002210000608

[51] Stein RB, Nichols TR, Jhamandas J, Davis L, Charles D (1977) Stable long-term recordings from cat peripheral nerves. Brain Res 128: 21–38. 871910 10.1016/0006-8993(77)90233-5

[52] Omura T, Sano M, Omura K, Hasegawa T, Doi M, et al (2005) Different expressions of BDNF, NT3, and NT4 in muscle and nerve after various types of peripheral nerve injuries. J Peripher Nerv Syst 10: 293–300. 16221288 10.1111/j.1085-9489.2005.10307.x

[53] Arvanian VL, Horner PJ, Gage FH, Mendell LM (2003) Chronic neurotrophin-3 strengthens synaptic connections to motoneurons in the neonatal rat. J Neurosci 23: 8706–8712. 14507970 10.1523/JNEUROSCI.23-25-08706.2003 PMC6740423

[54] Mentis GZ, Alvarez FJ, Shneider NA, Siembab VC, O'Donovan MJ (2010) Mechanisms regulating the specificity and strength of muscle afferent inputs in the spinal cord. Ann N Y Acad Sci 1198: 220–230. 20536937 10.1111/j.1749-6632.2010.05538.x PMC3027487

[55] Gonzalez M, Collins WF, 3rd (1997) Modulation of motoneuron excitability by brain-derived neurotrophic factor. J Neurophysiol 77: 502–506. 9120591 10.1152/jn.1997.77.1.502

[56] Boyce VS, Park J, Gage FH, Mendell LM (2012) Differential effects of brain-derived neurotrophic factor and neurotrophin-3 on hindlimb function in paraplegic rats. Eur J Neurosci 35: 221–232. 22211901 10.1111/j.1460-9568.2011.07950.x PMC3509221

[57] Ziemlinska E, Wewior I, Czarkowska-Bauch J, Kugler S, Bahr M, et al.. (2010) Adeno-associated viral vector-mediated BDNF overexpression in spinal rats counteracts GABA deficits both rostrally and caudally to the lesion and affects locomotion. Society for Neuroscience. San Diego.

[58] Davis-Lopez de Carrizosa MA, Morado-Diaz CJ, Tena JJ, Benitez-Temino B, Pecero ML, et al (2009) Complementary actions of BDNF and neurotrophin-3 on the firing patterns and synaptic composition of motoneurons. J Neurosci 29: 575–587. 19144857 10.1523/JNEUROSCI.5312-08.2009 PMC6664940

[59] Munson JB, Shelton DL, McMahon SB (1997) Adult mammalian sensory and motor neurons: roles of endogenous neurotrophins and rescue by exogenous neurotrophins after axotomy. J Neurosci 17: 470–476. 8987771 10.1523/JNEUROSCI.17-01-00470.1997 PMC6793713

[60] Wilson-Gerwing TD, Stucky CL, McComb GW, Verge VM (2008) Neurotrophin-3 significantly reduces sodium channel expression linked to neuropathic pain states. Exp Neurol 213: 303–314. 18601922 10.1016/j.expneurol.2008.06.002 PMC2751854

[61] Tauszig-Delamasure S, Yu LY, Cabrera JR, Bouzas-Rodriguez J, Mermet-Bouvier C, et al (2007) The TrkC receptor induces apoptosis when the dependence receptor notion meets the neurotrophin paradigm. Proc Natl Acad Sci U S A 104: 13361–13366. 17686986 10.1073/pnas.0701243104 PMC1948910

