# Correction: Enhancing Proprioceptive Input to Motoneurons Differentially Affects Expression of Neurotrophin 3 and Brain-Derived Neurotrophic Factor in Rat Hoffmann-Reflex Circuitry

**DOI:** 10.1371/annotation/196dc3ba-c963-46ee-a41d-2cb862ef2736

**Published:** 2014-01-16

**Authors:** Olga Gajewska-Woźniak, Małgorzata Skup, Stefan Kasicki, Ewelina Ziemlińska, Julita Czarkowska-Bauch

This article is missing the Supporting Information File "Text S1". Please use the following link to access Text S1: 
