# Supplementary figures and images for: Enhancing Proprioceptive Input to Motoneurons Differentially Affects Expression of Neurotrophin 3 and Brain-Derived Neurotrophic Factor in Rat Hoffmann-Reflex Circuitry
Source: PLoS One. 2013 Jun 11;8(6):e65937. doi: 10.1371/journal.pone.0065937 (PMC3679030; doi:10.1371/journal.pone.0065937)

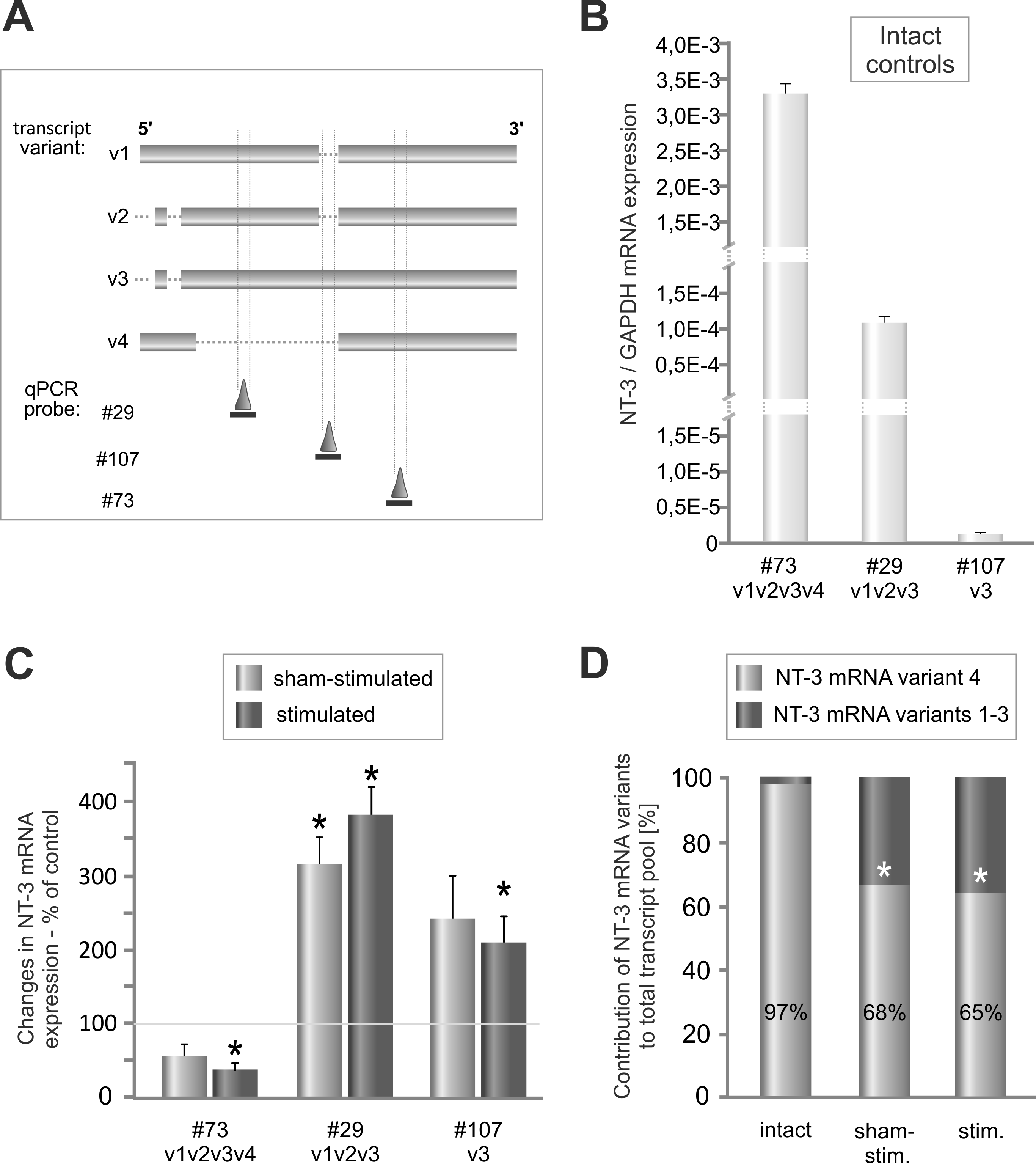

Supplement: Figure S1 — Contribution of NT-3 transcript variants to the total pool of NT-3 mRNA in the soleus muscle. A. Maps of 4 variants of NT-3 transcripts. Triangles point to sequences recognized by individual probes used in qPCR analysis. B. In the intact controls the level of the transcript variant 4 was 30 times higher than the level of three remaining variants (compare the results for probe #73 and #29). C. Electrical stimulation of the tibial nerve caused a significant down-regulation of the total mRNA level (probe #73) and similar tendency in the sham-stimulated side. A decrease is attributed to the abundant transcript variant 4 since three remaining variants (v1÷3) as well as variant 3 alone, analyzed separately, were significantly upregulated. D. By increasing the levels of v1÷3 transcripts and decreasing the level of v 4 transcript, electrical stimulation and sham-stimulation alters their contribution to an overall NT-3 mRNA pool. (TIF) [file pone.0065937.s001.tif]
