# Supplementary material for: Enhancing Proprioceptive Input to Motoneurons Differentially Affects Expression of Neurotrophin 3 and Brain-Derived Neurotrophic Factor in Rat Hoffmann-Reflex Circuitry
Source: PLoS One. 2013 Jun 11;8(6):e65937. doi: 10.1371/journal.pone.0065937 (PMC3679030; doi:10.1371/journal.pone.0065937)
Supplement: Table S1 — Sequences of TaqMan probes and primers used for quantitative analysis of NT3, BDNF, TrkC and TrkB gene expression by qPCR. (DOC) [file pone.0065937.s002.doc]

| **Gene** | **Transcript variant** | **NCBI Reference Sequence** | **UPL TaqMan Probe** | **Forward Primer (5’ – 3’)** | **Reverse Primer (5’ – 3’)** | **Amplicon location** |
| --- | --- | --- | --- | --- | --- | --- |
| ***NT3*** | 1  2  3  4 | NM_031073.3 NM_001270869.1 NM_001270868.1  NM_001270870.1 | #73  TCCTCAGC | AGTGAGAGCCTGTGGGTGAC | TAACCTGGTGTCCCCGAAT | Located in the coding exon II of NT3 gene thus makes no distinction between transcript variants. Synthesis of all three isoforms of NT3 protein is possible. |
|  | 1  2  3 | NM_031073.3 NM_001270869.1 NM_001270868.1 | #29  CTTCTGCC | CAGCGTCCCTGGAAATAGTC | TGGACATCACCTTGTTCACCT | Located in alternatively spliced noncoding exon I C (Kendall et al., 2000), this sequence is common for transcript variants 1, 2 and 3. |
|  | 3 | NM_001270868.1 | #107  TGCTGGGC | CTTCTGCCACGGCTCACC | GGACATCACCTTGTTCACCTG | PCR amplicon is located at the very beginning of the 5’ coding exon II (Kendall et al., 2000). Amplified sequence is unique for variant 3 (37 nt) and not present in the three other variants. Variant (3) differs also in the 5' UTR region as compared to variant 1 and 4. The resulting isoform of the NT3 protein (2) is of the same size but has a distinct N-terminus, compared to isoform 1. |
| ***TrkC.FL*** | 1 (TrkC.FL_14_)  2 (TrkC.FL_25_)  3 (TrkC.FL) | NM_001270656.1  NM_001270655.1  NM_019248.2 | #113  TCTGCCCA | TGGTTCCAGCTCTCTAACACAG | CCCCAGCATGACATCATATACTT | Located in region coding for intracellular catalytic domain of full-length TrkC receptor, in close proximity to the substrate binding site and tyrosine residue activation loop.  Gives rise to three full-length TrkC isoforms which differ in the presence (TrkC.FL_14_ and TrkC.FL_25_) or absence (TrkC.FL) of sequences inserted into the tyrosine kinase domain (Lamballe et al., 1993). |
| ***BDNF*** | 1  2  3  4  5  6  7  8  9  10 | NM_001270630.1  NM_012513.4  NM_001270631.1  NM_001270632.1  NM_001270633.1  NM_001270634.1  NM_001270635.1  NM_001270636.1  NM_001270637.1  NM_001270638.1 | #67  TGCTGGAG | GCAGTCAAGTGCCTTTGGAG | CGGCATCCAGGTAATTTTTG | Located in the proBDNF region of the coding exon of the BDNF gene, thus made no distinction between transcript variants. |
| ***TrkB.FL*** | 1 (TrkB.FL) | M55291.1  NM_012731.2 | #106  AGCCAGAG | TGGGAAATGGAAACCAGAAG | TGGGTTTTCCTATGCAGGAC | Located in 3’ noncoding region of TrkB gene, not present in truncketed TrkB transcripts. |
