# Supplementary material for: Correction: Enhancing Proprioceptive Input to Motoneurons Differentially Affects Expression of Neurotrophin 3 and Brain-Derived Neurotrophic Factor in Rat Hoffmann-Reflex Circuitry
Source: PLoS One. 2014 Jan 16;9(1):10.1371/annotation/196dc3ba-c963-46ee-a41d-2cb862ef2736. doi: 10.1371/annotation/196dc3ba-c963-46ee-a41d-2cb862ef2736 (PMC3894305; doi:10.1371/annotation/196dc3ba-c963-46ee-a41d-2cb862ef2736)
Supplement: Supplementary file 1 [file pone.196dc3ba-c963-46ee-a41d-2cb862ef2736.s001.pdf]

Contribution of NT-3 transcript variants to the total pool of NT-3 mRNA in the soleus muscle

To find the source of reduced NT-3 mRNA level in the soleus muscle, which was not reflected by changes in NT-3 protein, upregulated by stimulation, we attempted to evaluate a contribution of four NT-3 transcript variants (v1÷v4) in an overall changes of NT-3 mRNA expression. The assumption was that the complex structure of the rat NT-3 gene could control NT-3 protein synthesis by governing its multifaceted temporal and spatial mRNA expression, similarly as it was shown for BDNF [1,2].

The NT-3 gene consists of two small noncoding 5' exons (exons I A and I B) and a larger downstream 3' exon that encodes the NT-3 protein (exon II). The sequences of noncoding 5' exons and their alternative splicing play a crucial role in the translation, degradation and cellular localization of gene products. Two main classes of NT-3 transcripts are generated by alternative splicing of exon I A or exon I B, which are regulated by separate promoters, to the common exon II [3]. Small exon IC, located between exon IB and II [4,5], multiple transcription start sites in both 5' exons, and three different polyadenylation sites [3], multiply possible number of NT-3 mRNA variants and give a picture of intricate NT-3 gene regulation. Transcript variant 4 (Fig. S1 A) codes the shortest NT-3 protein isoform 3 (UniProtKB/Swiss-Prot: P18280, 258 aa). Its abundance suggests its functional importance. Few reports on region-specific expression profiles of each of the adult rat 5' exon-specific transcripts are available [4,5] but there is no data on their regulation and, in particular, on the impact of pattern and intensity of neuronal activation on NT-3 transcript variants. Experimental studies to date used primers derived from the exon II of the NT-3 gene, making no distinction between transcript variants [6,7,8] and therefore omitting an analysis of NT-3 gene expression at the post-transcriptional level.

Although in all tissues of the H-reflex circuitry we were able to detect all transcript variants, the level of variant 4 (devoid of exon I B) constituted 97% of all transcripts in the intact controls (as calculated by subtracting the signal intensity of 1÷3 variants from the signal derived from 1÷4 variants as there is no possibility to directly assay the level of v4), thus contributing significantly to the total NT-3 transcript level (Fig. S1 B, compare results for probe #73 and #29). Our results are in line with those by Sekimoto et al., 1998 and Kendall et al., 2000, who showed that the expression of exon I B - containing transcripts is low both in the spinal cord and sciatic nerve, in contrast to I A containing transcripts, which are abundant in both tissues [4,5].

Since an analysis of structural differences between transcripts 1÷4 (Table S1) indicates the variability in the N-terminal part of the protein which might control NT-3 subcellular location and/or secretion, an alternative splicing may be the mechanism affecting the regulation of NT-3 levels and its extracellular availability. Indeed, after stimulation, the

expression pattern of the minor transcripts pool, consisting of variants 1, 2 and 3 (Fig. S1 C, probe #29), which give rise to NT-3 isoforms 1 and 2 with extended N-terminal sequences, resembled the course of changes in total NT-3 protein level (Fig. 4 B). At the same time, transcript variant 4 was significantly down-regulated (Fig. S1 C, compare the results for probe #73 and #29) and, due to its high abundance, overshadowed the changes in transcripts 1÷3. As a consequence, both in sham- and stimulated groups, the contribution of variant 4 in the total pool of transcripts decreased significantly (by 32 and 35%, respectively, $p<0.05$, Wilcoxon test), suggesting that at least in part, increased NT-3 protein in the soleus muscle is due to its translation from v1÷3 transcripts (Fig. S1 D). To clarify the biological significance of activity-dependent code for generation of NT-3 transcript variants and isoforms of NT-3 protein, detailed studies are necessary, like those conducted to establish patterns of BDNF expression [1,2,10,11].

References:

1. Timmusk, T., Palm, K., Metsis, M., Reintam, T., Paalme, V., Saarma, M., Persson, H (1993) Multiple promoters direct tissue-specific expression of the rat BDNF gene. Neuron 10, 475–489.
2. Baj G, Leone E, Chao MV, Tongiorgi E (2011) Spatial segregation of BDNF transcripts enables BDNF to differentially shape distinct dendritic compartments. PNAS 108(40):16813-8.
3. Leingartner A, Lindholm D (1994) Two promoters direct transcription of the mouse NT-3 gene. Eur J Neurosci 6: 1149-1159.
4. Sekimoto M, Fukamachi K, Nemoto F, Miyata S, Degawa M, et al. (1998) Novel alternative splicing in the 5' exon of the neurotrophin-3 gene. Neuroreport 9: 3675-3679.
5. Kendall S, Yeo M, Henttu P, Tomlinson DR (2000) Alternative splicing of the neurotrophin-3 gene gives rise to different transcripts in a number of human and rat tissues. J Neurochem 75: 41-47.
6. Katoh-Semba R, Takeuchi IK, Inaguma Y, Ito H, Kato K (1999) Brain-derived neurotrophic factor, nerve growth and neurotrophin-3 selected regions of the ratbrain following kainic acid-induced seizure activity. Neurosci Res. 35(1):19-29.
7. Gomez-Pinilla F, Ying Z, Roy RR, Hodgson J, Edgerton VR (2004) Afferent input modulates neurotrophins and synaptic plasticity in the spinal cord. J Neurophysiol 92: 3423-3432.
8. Cote MP, Azzam GA, Lemay MA, Zhukareva V, Houle JD (2011) Activity-dependent increase in neurotrophic factors is associated with an enhanced modulation of spinal reflexes after spinal cord injury. J Neurotrauma 28: 299-309.
9. Lamballe F, Tapley P, Barbacid M (1993) trkC encodes multiple neurotrophin-3 receptors with distinct biological properties and substrate specificities. EMBO J 12: 3083-3094.
10. Aid T, Kazantseva A, Piirsoo M, Palm K, Timmusk T (2007) Mouse and rat BDNF gene structure and expression revisited. J Neurosci Res 85(3):525-535.
11. Tongiorgi E (2008) Activity-dependent expression of brain-derived neurotrophic factor in dendrites: facts and open questions. Neurosci Res. 61(4):335-46.
